# Reply to Muralidhar et al., Kenny et al., and Hotz et al.: The benefits of engagement with external research teams

**DOI:** 10.1073/pnas.2401501121

**Published:** 2024-03-05

**Authors:** Ron S. Jarmin, John M. Abowd, Robert Ashmead, Ryan Cumings-Menon, Nathan Goldschlag, Michael Hawes, Sallie Ann Keller, Daniel Kifer, Philip Leclerc, Jerome P. Reiter, Rolando A. Rodríguez, Ian Schmutte, Victoria A. Velkoff, Pavel I. Zhuravlev

**Affiliations:** ^a^Office of the Deputy Director, United States Census Bureau, Washington, DC 20233; ^b^Department of Economics, Cornell University, Ithaca, NY 14853; ^c^Department of Statistical Science, Duke University, Durham, NC 27708; ^d^Department of Economics, The University of Georgia, Athens, GA 30602

We thank Kenny et al. (1), Hotz et al. (2), and Muralidhar et al. (3) for taking the time to comment on our recent paper [Jarmin et al. (4)]. The modernization of disclosure avoidance technology at the Census Bureau has elicited substantial response from academia, and we are delighted to see that the letters to the editor exhibit the passion that the authors hold for their research and for census data. Feedback from these and other scholars resulted in tangible and notable improvements to the 2020 Census Disclosure Avoidance System, and we are confident that continued discussion and debate on these issues will serve to further benefit the Census Bureau and its statistical products moving forward. For that, we are extremely grateful.
